# An Assisted Diagnosis Model for Cancer Patients Based on Federated Learning

**DOI:** 10.3389/fonc.2022.860532

**Published:** 2022-03-03

**Authors:** Zezhong Ma, Meng Zhang, Jiajia Liu, Aimin Yang, Hao Li, Jian Wang, Dianbo Hua, Mingduo Li

**Affiliations:** ^1^ Hebei Engineering Research Center for the Intelligentization of Iron Ore Optimization and Ironmaking Raw Materials Preparation Processes, North China University of Science and Technology, Tangshan, China; ^2^ Hebei Key Laboratory of Data Science and Application, North China University of Science and Technology, Tangshan, China; ^3^ The Key Laboratory of Engineering Computing in Tangshan City, North China University of Science and Technology, Tangshan, China; ^4^ College of Science, North China University of Science and Technology, Tangshan, China; ^5^ Tangshan Intelligent Industry and Image Processing Technology Innovation Center, North China University of Science and Technology, Tangshan, China; ^6^ Beijing Sitairui Cancer Data Analysis Joint Laboratory, Beijing, China; ^7^ State Key Laboratory of Process Automation in Mining and Metallurgy, Beijing, China; ^8^ Beijing Key Laboratory of Process Automation in Mining and Metallurgy, Beijing, China

**Keywords:** cancer, machine learning, federated learning, cancer recurrence, diagnostic model

## Abstract

Since the 20th century, cancer has been a growing threat to human health. Cancer is a malignant tumor with high clinical morbidity and mortality, and there is a high risk of recurrence after surgery. At the same time, the diagnosis of whether the cancer is *in situ* recurrence is crucial for further treatment of cancer patients. According to statistics, about 90% of cancer-related deaths are due to metastasis of primary tumor cells. Therefore, the study of the location of cancer recurrence and its influencing factors is of great significance for the clinical diagnosis and treatment of cancer. In this paper, we propose an assisted diagnosis model for cancer patients based on federated learning. In terms of data, the influencing factors of cancer recurrence and the special needs of data samples required by federated learning were comprehensively considered. Six first-level impact indicators were determined, and the historical case data of cancer patients were further collected. Based on the federated learning framework combined with convolutional neural network, various physical examination indicators of patients were taken as input. The recurrence time and recurrence location of patients were used as output to construct an auxiliary diagnostic model, and linear regression, support vector regression, Bayesling regression, gradient ascending tree and multilayer perceptrons neural network algorithm were used as comparison algorithms. CNN’s federated prediction model based on improved under the condition of the joint modeling and simulation on the five types of cancer data accuracy reached more than 90%, the accuracy is better than single modeling machine learning tree model and linear model and neural network, the results show that auxiliary diagnosis model based on the study of cancer patients in assisted the doctor in the diagnosis of patients, As well as effectively provide nutritional programs for patients and have application value in prolonging the life of patients, it has certain guiding significance in the field of medical cancer rehabilitation.

## Introduction

Since the 20th century, the improvement of information storage capacity and the continuous improvement of information processing speed have promoted the rapid development of the data storage industry and big data information technology, and at the same time produced a huge thrust for the birth and development of emerging industries. At present, the amount of data output in the medical field is increasing exponentially. Through effective data resource storage and transmission management technology, combined with big data mining technology, the utilization efficiency and intelligence of data in the medical field have been improved (1, 2), giving medicine rapid development of the field has injected new impetus. This paper studies the influencing factors of postoperative recurrence of cancer patients, and proposes a federated learning model suitable for predicting the auxiliary diagnosis and prediction of cancer patients. The clinical data of patients is collected and combined with the prediction model to predict the location of cancer recurrence in recovered patients. Further Assisting doctors in diagnosis and improving the survival rate of cancer patient’s has certain significance in the fields of cancer care, rehabilitation and clinical diagnosis.

With the continuous improvement of the material living standard of human society, the living environment and lifestyle of human beings have also changed correspondingly. The cancer problem that comes with it has become one of the most serious problems threatening human health. According to the International Agency for Research on Cancer According to the estimated data of “Global Cancer Incidence and Mortality in 2018” (GLOBOCAN2018) (3), there were approximately 18.1 million new cancer cases and 9.6 million cancer deaths worldwide in 2018. In 1971, the United States first proposed the concept of “tumor rehabilitation” (4), the main purpose of which is to help cancer patient’s recover their mental, physical and physical functions under cancer conditions and limited treatment. The way of cancer rehabilitation mainly depends on the nature of the tumor and the stage of development of the tumor (5). Early detection and rehabilitation of cancer have greatly improved the survival rate and quality of life of patients. However, the factors affecting cancer patient’s’ recurrence after surgery are complex, so it is very challenging to predict the condition and trend of cancer patient’s after surgery. In this regard, many scholars have done some work. Based on the evaluation data of cancer patients, some scholars have classified and predicted benign or malignant tumors, predicted postoperative recurrence time, and predicted the type of tumor. And trend research (6–8), the continuous development of machine learning and deep learning fields has also played a huge role in assisting cancer diagnosis and treatment (9, 10). However, there are two problems in the development of machine learning technology. On the one hand, data security is difficult to guarantee, and privacy protection issues are becoming more and more serious. On the other hand, because data sharing has become a new trend, and in order to prevent leakage of data among enterprises, data protection has been strengthened, and data in the era of big data has been reduced. Sharing, machine learning has encountered obstacles in data sharing training, resulting in the phenomenon of “data islands” (11). In the medical environment, the phenomenon of data islands also exists among hospitals. In order to break the phenomenon of “data islands”, Google proposed the concept of federated learning (12) in 2016, which was originally used to solve Android mobile terminals. The problem of users updating the model locally, the design goal is to carry out between multiple parties or multiple computing nodes under the premise of ensuring information security during big data exchange, protecting terminal data and personal data privacy, and ensuring legal compliance and efficient machine learning. Among them, the machine learning algorithms that can be used in federated learning are not limited to neural networks, but also include important algorithms such as random forests. This model effectively solves the problem of privacy protection during data sharing between various enterprises. This model not only improves the security of data sharing between enterprises, on the other hand, because of the data sharing between enterprises, the accuracy of the training model also increases. At the same time, diagnosing whether the cancer is recurring *in situ* and predicting the time of recurrence are crucial for the patient’s next rehabilitation treatment. According to statistics, about 90% of cancer-related deaths are caused by failure to prepare for cancer recurrence and cancer cell metastasis. Therefore, research on accurately predicting the location of cancer recurrence and resetting time under the premise of ensuring data security is for cancer Clinical diagnosis and treatment are of great significance.

## Auxiliary Diagnosis Model Construction Related Work

At present, cancer has always been a worldwide medical problem. With the gradual increase in the incidence of cancer, traditional cancer rehabilitation forecasts and cancer rehabilitation programs given by doctors through their own experience can no longer meet the needs of patients, and cancer is generally difficult to achieve a complete cure. The effect of cancer treatment is often limited to improving symptoms, with the goal of improving the quality of life of patients during the survival period and prolonging life span. Although cancer patients cannot be completely cured, more and more advanced technologies are applied in the medical field. The current 5-year survival rate of patients with advanced cancer has increased from 2%~5% decades ago to 16%~23% today. In the future, the accumulation of cancer patient data and the vigorous development and application of artificial intelligence will have more advantages than traditional medical models. In the long run, the application of artificial intelligence will surely drive the field of cancer rehabilitation diagnosis to high-end, personalized, precise, and intelligent. This research will adopt the convolutional neural network algorithm based on the federated learning framework to predict the recurrence time and location of cancer patients. On the one hand, federated learning has sufficient guarantee for the safety of cancer patient data. On the other hand, cancer patient’s data is predicted under the federated learning framework, which provides a guarantee for the safety of patient data among hospitals, greatly increases the amount of training data, and makes the training model more accurate in the end, which benefits multiple parties. Finally, the doctor outputs the results and patient information through the model. Analysis of the correlation degree, timely intervention in the rehabilitation process of patients, in order to improve the survival time of patients.

### Federated Learning

In the context of the gradual maturity of machine learning and the realization of automatic identification and intelligent decision-making, in order to solve the problem of data privacy protection, federated learning (13–16) emerged as a potential solution.

Since the training data is still stored locally in the participants during the federated learning process, this mechanism can not only realize the sharing of the training data of each participant, but also ensure the protection of the privacy of each participant (17). The basic workflow of federated learning mainly includes:

1. Participants download the initialized global model from the cloud server, use the local data set to train the model, and generate the latest local model update (model parameters).
2. The cloud server collects various local update parameters and updates the global model through the model averaging algorithm. Because of the unique advantage of federated learning-a unified machine learning model can be trained from the local data of multiple participants under the premise of protecting data privacy.

**Figure 1:**
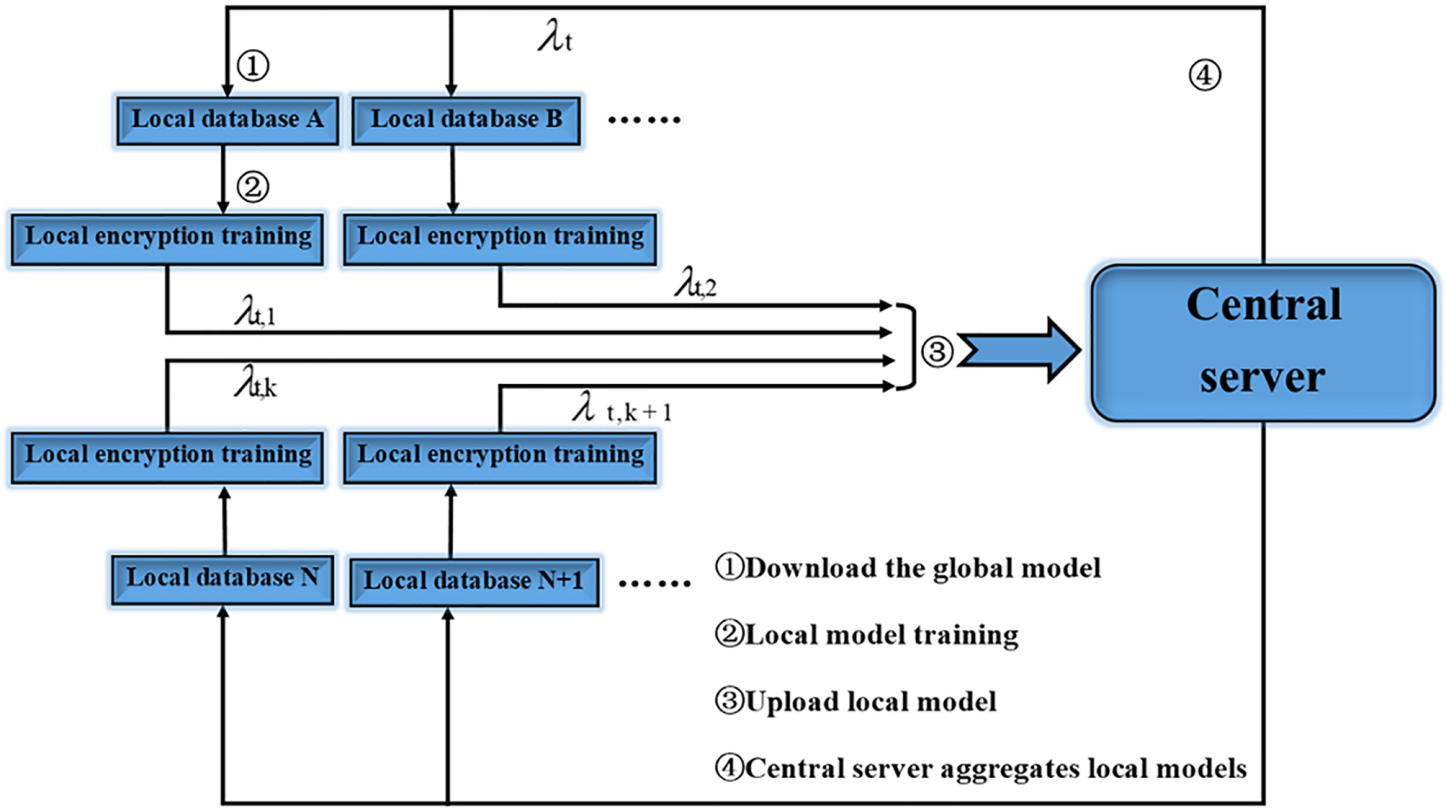
Federated learning workflow.

Its main innovation is to provide a distributed machine learning framework with privacy protection features. Its working principle is as shown in **Figure 1**, and it can cooperate with thousands of participants in a distributed manner for a specific machine learning model. Iterative training, an iterative process of federated learning is as follows:

1. The client downloads the global model *λ*_t, k+1_ from the server.
2. Client *k* trains local data to obtain local model *λ*_t, l_
3. Clients of all parties upload local model updates to the central server.
4. The server performs a weighted aggregation operation after receiving the data from all parties to obtain the global model *λ*_t_.

Among them, represents the local model update of the *t-th* round of communication of the *k-th* client, and represents the global model update of the *t-th* round of communication.

It can be seen from the introduction and flow chart that the federated learning technology has the following characteristics.

1. The original data participating in the federated learning is kept on the local client, and only the model update information is interacted with the central server, and there is no data transmission in plain text.
2. The model jointly trained by the participants of federated learning will be shared by all parties who contribute training data.
3. The final model accuracy of federated learning is similar to that of centralized machine learning, and the accuracy is stronger.
4. The higher the quality of the training data of the federated learning participants, the higher the global model accuracy.

Federated learning can be divided into three categories: Horizontal Federated Learning, Vertical Federated Learning, Federated Transfer Learning. Horizontal federated learning is essentially the union of samples. The scope of application is where there is a large overlap of participant data features and a small overlap of user data. The data that can be used for joint modelling training is that part of the data where both parties have the same data characteristics but the users are not identical. For the part of the data, the horizontal federated learning application scenarios are more extensive. For example, between banks A and B in the same region, their businesses are similar (features similar), but users are different (different samples). Another example is the patient data of Hospital A and Hospital B for a particular case, which is also perfectly suitable for horizontal federal learning. There is data A in data B. Under the framework of the federated horizontal learning model, the server only conducts joint training for the common features of data A and data B and the parameters are returned to the participants. For this study, we have a total of three hospitals participating together. We select the experimental data strictly in combination with the characteristics of horizontal federated learning, and finally establish the model under the condition of ensuring that the data of each hospital is protected.

### Localized Differential Privacy Protection Method

Differential privacy is a privacy definition first proposed by Cynthia Dwork in 2006 (18), which was developed in a specific scenario of statistical disclosure control. Differential privacy provides a kind of information theory security guarantee, so that the output result of the function is insensitive to any specific record in the data set.

Differential privacy can be divided into centralized differential privacy and localized differential privacy according to the different ways of data mobile phones. The two are different from the stages of differential data. Centralized differential privacy requires a trusted third party to collect data and perform data differential work in a unified manner. However, the current problem is that it is difficult to find a trusted third party in our lives. Therefore, in the context of federated learning, localized differential privacy can fit well with the encryption process required by the federated learning framework. The data is preprocessed using the idea of localized differential privacy, and then the federated learning framework is used for subsequent operations to fully improve the data. The safety of the user and the safety of the user.

Localized differential privacy can transfer the data privacy processing process to each participant in federated learning, and the participants will process and protect the data themselves, which will further improve the security of the data, which is defined as (19, 20): for any one Localized differential privacy function *f*(*x*), its domain (domain) is *Dom*(*f*), range (range) is *Ran*(*f*), for any input *x*, *x*′∈ *Dom*(*f*), output *y*∈ *Ran*(*f*), we call function *f* provides (*ξ*) -localized differential privacy protection, y is the final output, currently only When it meets:


(1) $$\mathrm{Pr}[f(x)=y]\leq e^{\xi }\mathrm{Pr}[f(x')=y]$$

In the above formula, *ξ* represents the privacy budget.

The concept of localized differential privacy is similar to the concept of federated learning. In fact, we have combined the idea of localized differential privacy in the realization of this research.

### Convolutional Neural Network

**Figure 2:**
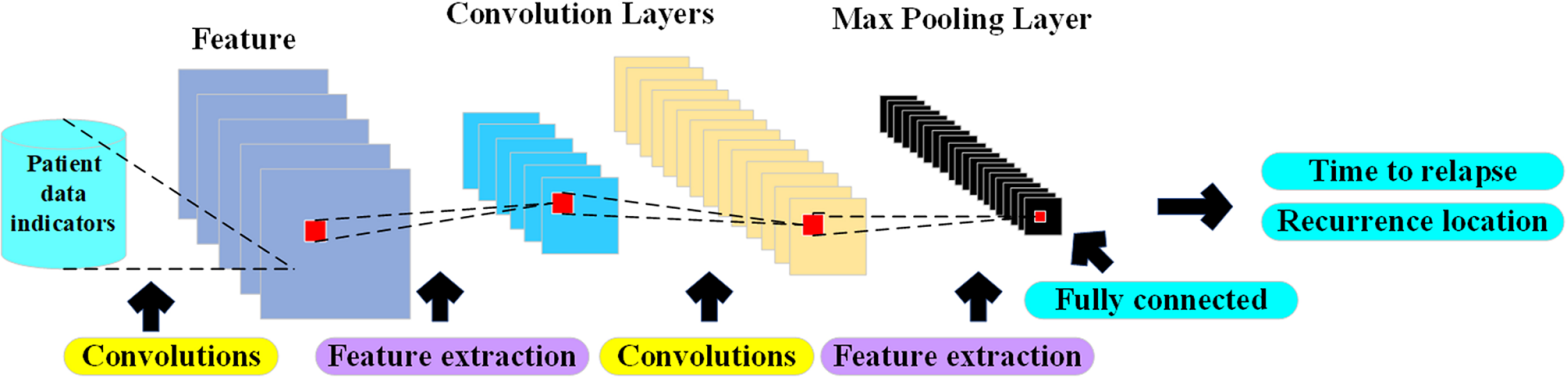
Auxiliary diagnosis CNN structure diagram.

CNN was proposed in 1998 by Yann LeCun of New York University (21). CNN is essentially a multilayer perceptron. The key to its success lies in the way it uses local connections and shared weights. On the one hand, it reduces the number of weights and makes the network easy to optimize, and on the other hand, it reduces overfitting risk (22). CNN is a kind of neural network. Its weight sharing network structure makes it more similar to biological neural network, which reduces the complexity of the network model and reduces the number of weights. The model structure of CNN is shown in **Figure 2**.

With the continuous increase in the incidence of cancer, the difficulty in detecting early cancer symptoms, and the various uncertainties in rehabilitation after cancer surgery, cancer has become the number one killer of human health today. In response to this difficulty and uncertainty, the CNN algorithm is widely used in cancer CT image detection and cancer postoperative recurrence prediction with its strong recognition ability and high prediction accuracy. The American artificial intelligence AI uses deep learning (CNN) to diagnose and treat cancer (23), and trains a deep convolutional neural network model to detect cancerous transformation of normal cells by letting artificial intelligence algorithms learn cancer CT images that far exceed the number of consultations of human doctors in a lifetime. The purpose is to achieve the purpose of early detection and early treatment.

In addition to showing high performance in recognizing cancer CT images, CNN also shows its powerful side in cancer prediction. As we all know, prostate cancer is the most common and the second most deadly cancer among American men. The classification of prostate cancer based on histological image Gleason classification is of great significance in patient risk assessment and treatment planning. In response to this problem, the regional convolutional neural network model was used to detect epithelial cells to predict the risk of cancer. After model training and experimental testing, the accuracy rate reached 99.8% (24).

In summary, CNN, as one of the deep learning algorithms, has mature theoretical foundations and experimental cases for cancer CT image detection and recognition or cancer incidence prediction, which provides theoretical guidance for the cancer rehabilitation medical recommendation system designed in this paper.

CNN is an artificial neural network with high recognition ability. In CNN, there are multiple neuron connections between each layer of the network. The convolution kernel is actually a user-defined size and weight matrix, which acts on the local perception domains in different regions of the same image, and extracts each local perception domain. And generate input values for the next layer of neurons. The convolutional layer convolves the input features, and the pooling layer reduces the size of the feature map through spatial invariance averaging or maximum operation. The activation function we use ReLU (22). The main advantage of CNNs is that they are easier to train and have fewer parameters than fully connected networks with the same number of hidden units. The feature map is shown in formula (2). The pooling layer performs secondary extraction of input features through specific pooling rules, and its feature map is shown in formula (3).


(2) $$H_{i}=f(H_{I-1}⊗\omega_{i}+b_{i})$$

(3) $$H_{j}=f(pooling(H_{I-1})+b_{j})$$

Among them, *H_i_* is the feature map, *f*(*x*) is a nonlinear activation function, “⊗“ is the convolution operation of the convolution kernel and the feature map, *ω* is the weight vector *b* is the bias, pooling (*x*) is a pooling rule, for example Average pooling layer, maximum pooling layer and random pooling layer.

The structure of the convolutional neural network designed in this paper takes into account the sample data as 6 indicators. The size of the convolutional layer is 3×3×128, 3×3×256, 3×3×512, and the pooling layer is uniformly designed to have a size of 2×2.

(1) Convolutional layer: the *j-th* feature image of the first layer is expressed as:


(4) $$X_{j}=g(\sum_{x_{i}\in M_{j}}x_{i}*k_{ij}+b_{j})$$

Among them, the nonlinear activation function *g*. The set of feature maps connected between the l-1th layer and the *j-th* feature map of the lth layer is denoted as *M_j_*, which means the set of input feature images. The offset is denoted as *b_j_*. The convolution kernel connecting the *i-th* feature map in the l-1 layer and the *j-th* map in the l-*th* layer is denoted as *k_ij_*

(2) Pooling layer: The pooling layer is denoted as the l-*th* layer, and the *j-th* feature map *x_j_* of the lth layer is expressed as:


(5) $$x_{j}=w_{j}pool(X_{j})+b_{j}$$

Among them, the weight coefficient *x_j_* = *w_j_ pool*(*X_j_*) + *b_j_* is denoted as *w_j_*, and the real number is taken in the general experiment. The bias is denoted as *b_j_*, and the pooling function is denoted as *pool*(). There are maximum pooling, average pooling, random pooling, and LP pooling.

(3) Fully connected layer: the output vector *x^l^* of the fully connected layer:


(6) $$x^{l}={\left(\beta^{l}\right)}^{T}v^{l-1}+b^{l}$$

Among them, the vector generated by the feature map of the pooling layer of the l-1 layer or the output vector of the feature map of the convolutional layer is denoted as *v^l^* ^–1^, the bias is denoted as *b^l^*, and the weight coefficient matrix is denoted as *β^l^*.

(As early as 2004, the Stanford University Medical and Mathematics Interdisciplinary Research of Cancer Patient Rehabilitation Intelligence Evaluation Model has been established, and it has shown its huge application prospects in clinical applications for hundreds of thousands of American cancer patients. In China, we In conjunction with the Stellite Cancer Data Analysis Laboratory, Hebei and Shanxi and other hospitals, it has long-term evaluated and tracked the rehabilitation process of thousands of cancer patients in China, analyzed their clinical data and rehabilitation data, and established Model of the system suitable for Chinese patients)

### Federated Learning Model Based on Convolutional Neural Network

In order to optimize the recurrence time and the accuracy of the recurrence location of cancer patients in the rehabilitation stage, and to solve the insufficient amount of data in a single hospital (25, 26), this question paper proposes a cancer patient-assisted diagnosis model that combines federated learning and convolutional neural networks. Use federated learning to protect user data privacy and expand the amount of data, and at the same time allow participants to collaborate to train a global model without sharing each other’s private data. For each participant, the local data needs to be pre-processed, including digitization, and standardized to convert the original data into a standard data format, and then the local data is the first step to protect the local data with local differential privacy.

The iterative process completes the training of parameters locally for the convolutional neural network model deployed by the third party, and the parameters include the convolution kernels and offset terms of each layer. Post-encryption training on patient data using homomorphic encryption, followed by uploading of parameters. After receiving the model parameters uploaded by the client, the server will iterate the model according to the configuration of the central server, update the parameters of the current model, and persist it for the next round of training parameter upload and aggregation before returning it to the participants. The iterative process of our overall model follows the basic federated learning iterative process, in which we fuse a convolutional neural network adapted to cancer patient data samples and configure the model to form continuous iterations. According to the data supply characteristics of each participant, we adopt the rules of horizontal federated learning and unify the data standards.

In actual training, we consider that each participating hospital only exchanges encrypted correlation coefficients with the server. This experiment is based on the case where the data scale of each hospital is equal or the difference is not large. The training model algorithm is as follows: **Algorithm 1** Shown.

**Algorithm 1:** CNN-FL model based on local differential privacy

1 For Iteration t do
/* Service-Terminal: * /
2 $\omega_{t}=\frac{1}{Q}\sum_{q=1}^{Q}\omega_{t}^{k};$
3 Send ω_t_ to each participant;
/* participants: * /
4 for Participant *q* do
5 Do Localized differential privacy
6 $\omega_{t}^{q}=\omega_{t};$
7 For Local epoch e do
8 $\omega_{t}^{q}=\omega_{t}^{q}-\eta \frac{\partial }{\partial X^{q}}Z℘$
9 End
10 End
11 End

Among them, *Q* represents the participant of *Q*; *ω_t_* represents the global model parameter during iteration *t*; $\omega_{t}^{k}$ represents the model parameter of the *q* th participant at iteration *t*; *η* represents the learning rate; *X^k^* represents the training of the q-*th* participant data set. It should be noted that differential privacy protection is added to the user side of the algorithm, and after a partial differential privacy model is initially formed, it constitutes a global model for user homogenization upload parameter training.

**Figure 3:**
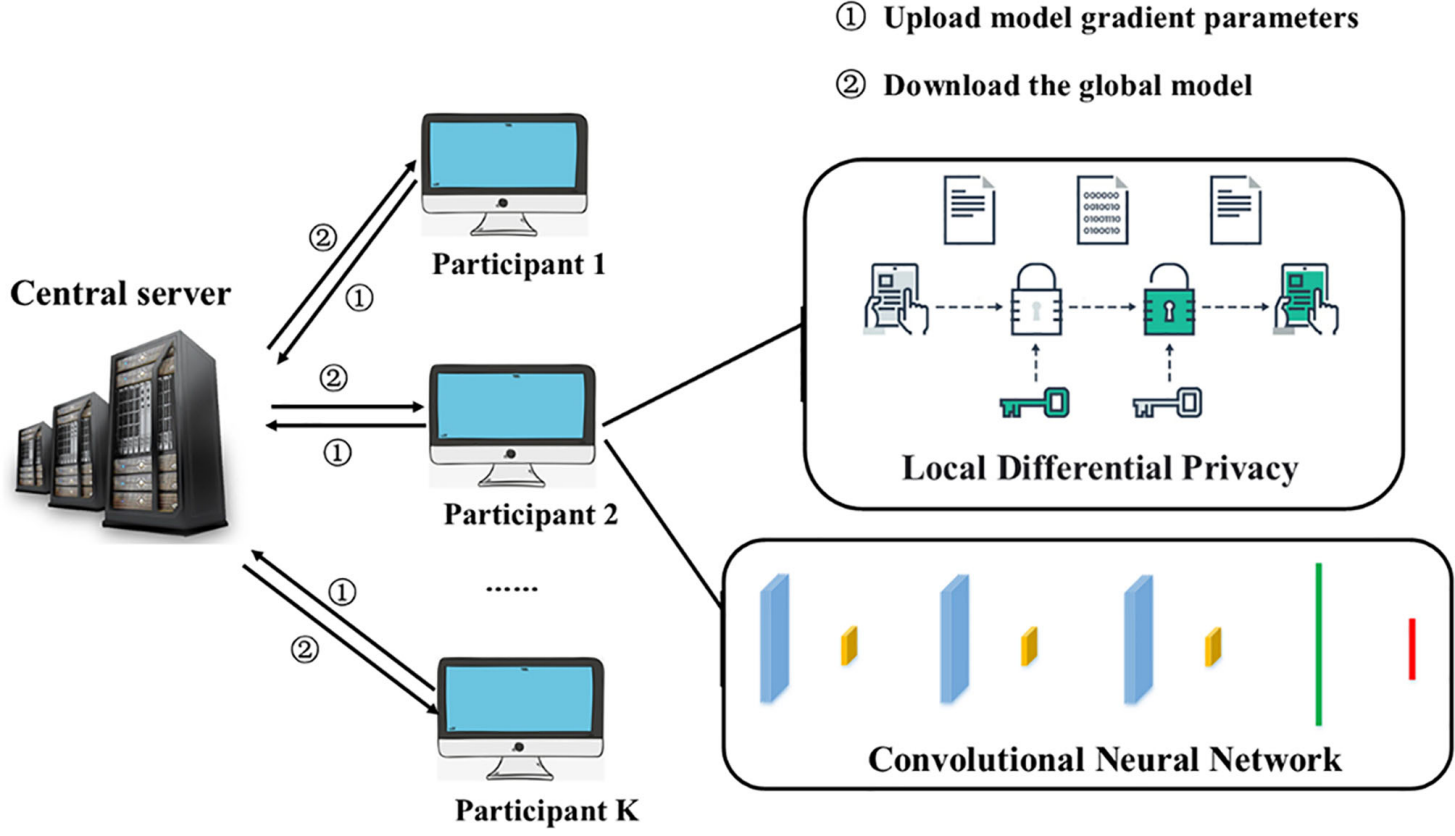
CNN-FL model structure.

As shown in Algorithm 1 above, each participant needs to use the local data set to train the CNN model. The model is shown in **Figure 3** below. In each iteration, each model participant first uploads the current model correlation coefficient to the server. The global model is updated by averaging the latest correlation coefficients of each participant. In the next iteration, each participant downloads the latest global model parameters and uses local data to train the CNN model. Iterate continuously until the overall model is optimal.

The current CNN-FL model has 100 participants. After 50 iterations, the global model parameter is *ω*_50_. At the 51st iteration, each participant’s initial local model parameter $\omega_{51}^{k}=\omega_{50}$ (the value interval of *k* is [1, 100]), After 51 iterations, the global model is updated to $\omega_{51}=\frac{1}{100}\sum_{k=1}^{100}\omega_{51}^{k}$ .

## Construction of Rehabilitation Data Sample Set Based on Federated Learning

According to the characteristics of federated learning and long-term medical consensus (27, 28), ASC carcinogenic factor research report and TIES.IO cancer assessment data, 12 factors that affect cancer recurrence are comprehensively selected: gender, age, basic score, tumor score, immune score, basic Nutrition score, nutritional comparison score, safe intake score, total nutrition score, microenvironment score, psychological score, aerobic activity score, collect data from cancer patients and score, use statistical correlation coefficient Pearson correlation coefficient, Spearman correlation coefficient to compare the sample Enter the indicators for correlation research, and finally determine 6 influencing factors: tumor score, immune score, basic nutrition score, psychological score, microenvironment score, aerobic exercise and advanced homework. The Pearson correlation coefficient is shown in **Table 1**. The related items of each index score and their weights are shown in **Table 2**. The weights are given based on the experience of doctors and experts (29). The data set in this article was collected from Shanxi Provincial People’s Hospital and Hebei Tumor Hospital, etc., a collaborative experiment in Beijing, China The office is responsible for cancer data evaluation and data processing, as well as liaison with various hospitals.

**Table 1:** Cancer Patient Index Evaluation Criteria.

Finger syndrome	Related terms and Weights
Immune score	CD3+CD4+CD8+/CD45+ (4); CD3+CD4+/CD45+ (8); CD4+/CD8+ (10); CD3+CD16+CD56+/CD45+ (6); CD3-CD56+ (5); CD4+CD25+ (1); Exercise ECG (X ± SD) (2); Sports Leather (X ± SD) (2).
Tumor score	Size (10); Placeholder (10); Violate the relationship (10); Angiogenesis (10); Pathological typing (3); CTC value (9); Differentiation (10); Mutation target (1).
Basic nutrition score	Total nutrition (6); Balanced nutrition (3); Nutrition safety assessment (5); Cancer cell proliferation (10); Immune cell proliferation (10); Angiogenesis (8); Amino acid evaluation (5); Proteomics evaluation (10).
Psychological score	Life event scale (1); Cornell Medical Index (2); Self-rating anxiety scale (5); Self-rating depression scale (5); Baker Anxiety Scale (5); Baker Depression Questionnaire (5); Pittsburgh sleep Quality index (4); Texas Social Behavior Questionnaire (3); Family function assessment (1); Exercise ECG (X ± SD) (2); Sports Leather (X ± SD) (2).
Microenvironment score	O2 (3); PH value (4); Interstitial pressure (2); Inflammatory response (7); Vascular permeability (6); CTC value (9); Proteomic analysis (8).
Exercise and advanced work	Aerobic exercise (4); Advanced social work (3); Texas Social Behavior Questionnaire (3).

**Table 2:** Correlation analysis table of each index.

Pearson correlation	Pearson-cor/Sig.	Imm	Tum	Mic	Heart	Nut	Aer
Imm	Pearson-cor	1	0.30^*^	0.04	0.17	0.21	-0.07
	Sig.		0.03	0.79	0.25	0.14	0.64
Tum	Pearson-cor	0.30^*^	1	0.09	-0.38^**^	0.10	0.03
	Sig.	0.03		0.54	0.01	0.48	0.86
Mic	Pearson-cor	0.04	0.09	1	0.21	-0.20	-0.003
	Sig.	0.79	0.54		0.14	0.18	0.99
Heart	Pearson-cor	0.17	-.038^**^	0.21	1	-0.15	-0.02
	Sig.	0.25	0.01	0.14		0.32	0.92
Nut	Pearson-cor	0.21	0.10	-0.20	-0.15	1	-0.19
	Sig.	0.14	0.48	0.18	0.32		0.19
Aer	Pearson-cor	-0.07	0.03	-0.003	-0.02	-0.19	1
	Sig.	0.64	0.86	0.99	0.92	0.19	

*At the 0.05 level (Sig.), the correlation is significant, **At the 0.01 level (Sig.), the correlation is significant.

It can be seen from **Table 2** that each index has a certain correlation, and we can see that the tumor index and the immune index are positively correlated, indicating that the stronger the immune index, the weaker the tumor index. Moreover, there is a negative correlation between psychological indicators and tumor indicators. The more ideal these indicators, the longer the patient will have to relapse.

## Experimental Simulation and Result Analysis

### Cancer Aided Diagnosis Model

**Figure 4:**
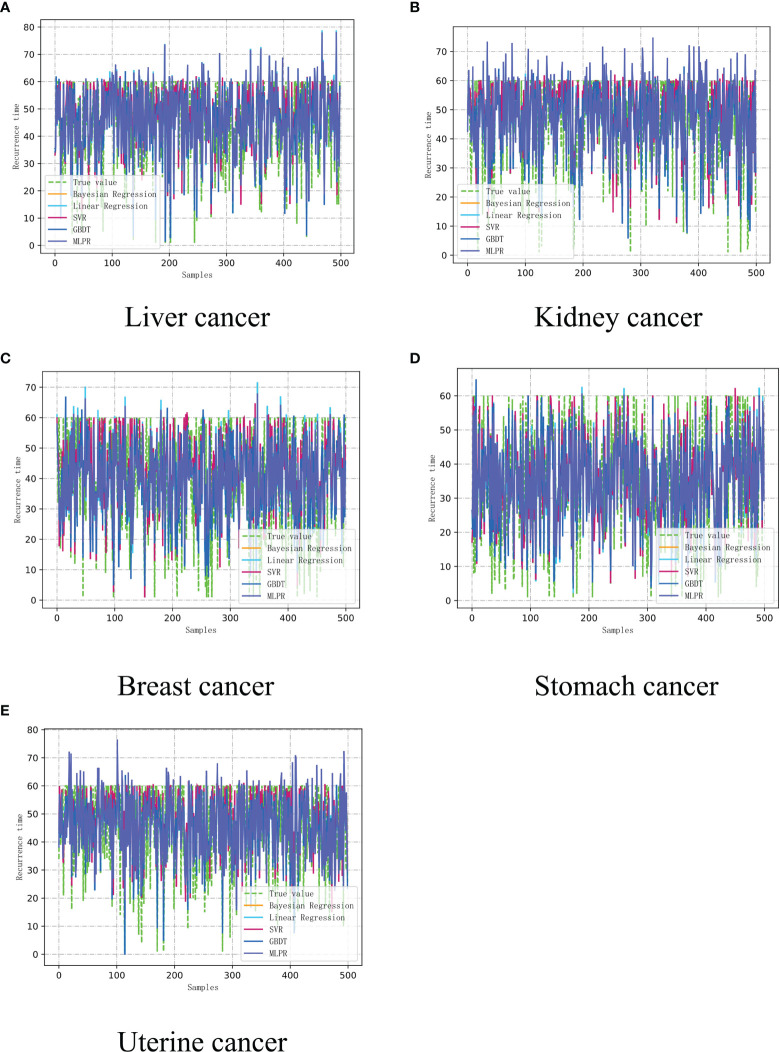
The recurrence time model of cancer assisted diagnosis based on machine learning. **(A)** Liver cancer. **(B)** Kidney Cancer. **(C)** Breast cancer. **(D)** Stomach cancer. **(E)** Uterine cancer.

We build a cancer-assisted diagnosis model. Based on the evaluation criteria of each indication described above, we trained and tested the machine learning-assisted diagnosis and treatment model on 500 sets of data (5 groups of 500 different cancer patient’s), and first established the input and output vectors, Among which sample input and output: the six major indicators of immune indication, tumor indication, microenvironment indication, psychological indication, nutrition indication, aerobic exercise and advanced homework as input, the predicted recurrence time and recurrence position As an output, the experimental results are shown in **Figure 4** below. We combined medical knowledge and intelligent diagnosis and treatment models to set the prediction error range of cancer recurrence time to ±6 months. During the experiment, we used the linear model of machine learning, the tree model, and the neural network of the multi-layer perceptron (MLP) in deep learning is used to test the cancer-assisted diagnosis model.

**Figure 5:**
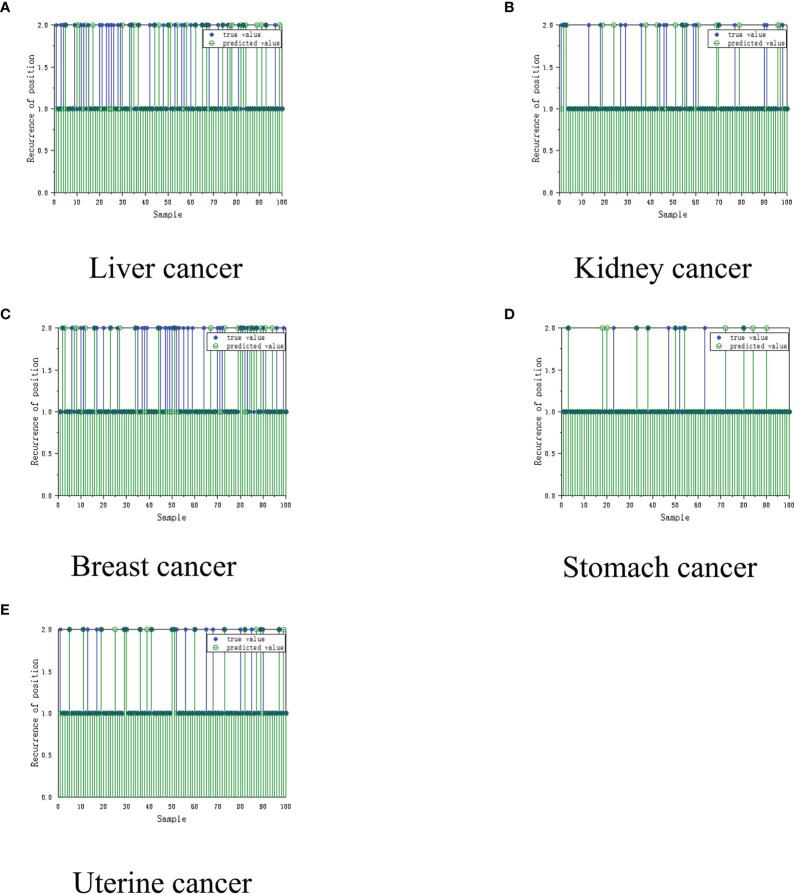
Recurrence location model for cancer assisted diagnosis based on machine learning. **(A)** Liver cancer. **(B)** Kidney Cancer. **(C)** Breast cancer. **(D)** Stomach cancer. **(E)** Uterine cancer.

Based on multiple iterative experiments and multiple experiments, combined with the absolute error of the cancer recurrence time, the accuracy of the prediction of the recurrence time of the five types of cancer patient’s is between 65%-85%. This result proves that the model has practical application value. The doctor can complete the diagnosis and treatment of the patient based on the results and refer to the various indicators of the patient, and this result is only completed in a unilateral modeling situation with a limited amount of data, and then we can determine whether the recurrence of the cancer patient has metastasized to another location, where 1.0 is The original position, 2.0 is that the cancer cells have metastasized. The test was performed using the constructed auxiliary diagnosis model. The test results of the neural network using the multi-layer perceptron (MLP) are shown in **Figure 5**.

It can be clearly seen from the figure above that the MLP network has been trained and tested on 100 sets of samples. The final performance of the MLP network is 90%, and the algorithm that predicts the location of cancer recurrence (that is, whether the cancer cell has metastasized to other parts of the body) can reach 90%. It was unstable. We subsequently simulated the patients, and showed excellent application prospects under the condition of unilateral machine learning modeling and insufficient data.

From the **Table 3**, we know whether the cancer cells of the patient in the sample have metastasized and whether they have recurred in situ. By predicting simulation and simulation, only one-way machine learning modeling can achieve very impressive results. Then, we proposed a method A convolutional neural network-assisted diagnosis model based on federated learning. On the one hand, this model protects the data privacy of patients. On the other hand, through the joint modeling of multiple hospitals, they can share each other’s data but protect each other’s privacy.

**Table 3:** Unilateral modeling and simulation of recurrence location.

MLP neural network	Liver cancer	Kidney Cancer	Breast cancer	Stomach cancer	Uterine cancer
In-situ (simulation)	60%	77%	61%	91%	86%
Transfer (simulation)	40%	23%	39%	9%	14%
*In situ* (actual)	62%	80%	60%	89%	76%
Transfer (actual)	38%	20%	40%	11%	24%

### Convolutional Neural Network Aided Diagnosis Model Based on Federated Learning

**Figure 6:**
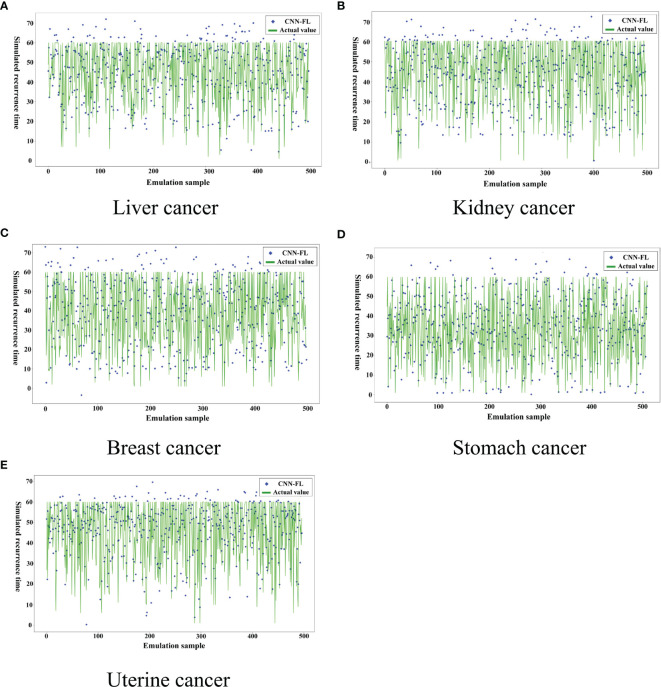
Recurrence time model of cancer assisted diagnosis based on federated learning.

We analyze the disadvantages of data processing of cancer patient’s based on machine learning. Among them, machine learning adopts unilateral modeling, and the data is unprotected. The amount of data in a single hospital is not sufficient, but it still achieves good results, but diagnosis and treatment based on machine learning The model is difficult to truly enter the application level and faces many data security issues. Therefore, we established a convolutional neural network-assisted diagnosis model based on federated learning, built the FL-CNN model based on the federated learning framework, and used privacy protection methods. The model parameter transmission updates the model, and after several rounds of parameter updates, we analyze the cancer recurrence time and the accuracy rate of the recurrence location in five types of cancer patients. The data volume used under the federated learning framework (after differential privacy) is shown in **Table 4** below, based on the federation The experimental results of the learned convolutional neural network cancer recurrence time simulation model are shown in **Figure 6** below.

**Table 4:** Introduction to the data set of participants.

Overall model data information (after localized differential privacy)	Hebei A Hospital, China	Shanxi B Hospital, China	Beijing C Hospital, China
Liver cancer	800-900 (1000)	800-900 (1000)	800-900 (1000)
Kidney Cancer	300-350 (400)	300-350 (400)	300-350 (400)
Breast cancer	300-350 (400)	300-350 (400)	300-350 (400)
Stomach cancer	175-215 (250)	175-215 (250)	175-215 (250)
Uterine cancer	200-250 (300)	200-250 (300)	200-250 (300)

**Figure 7:**
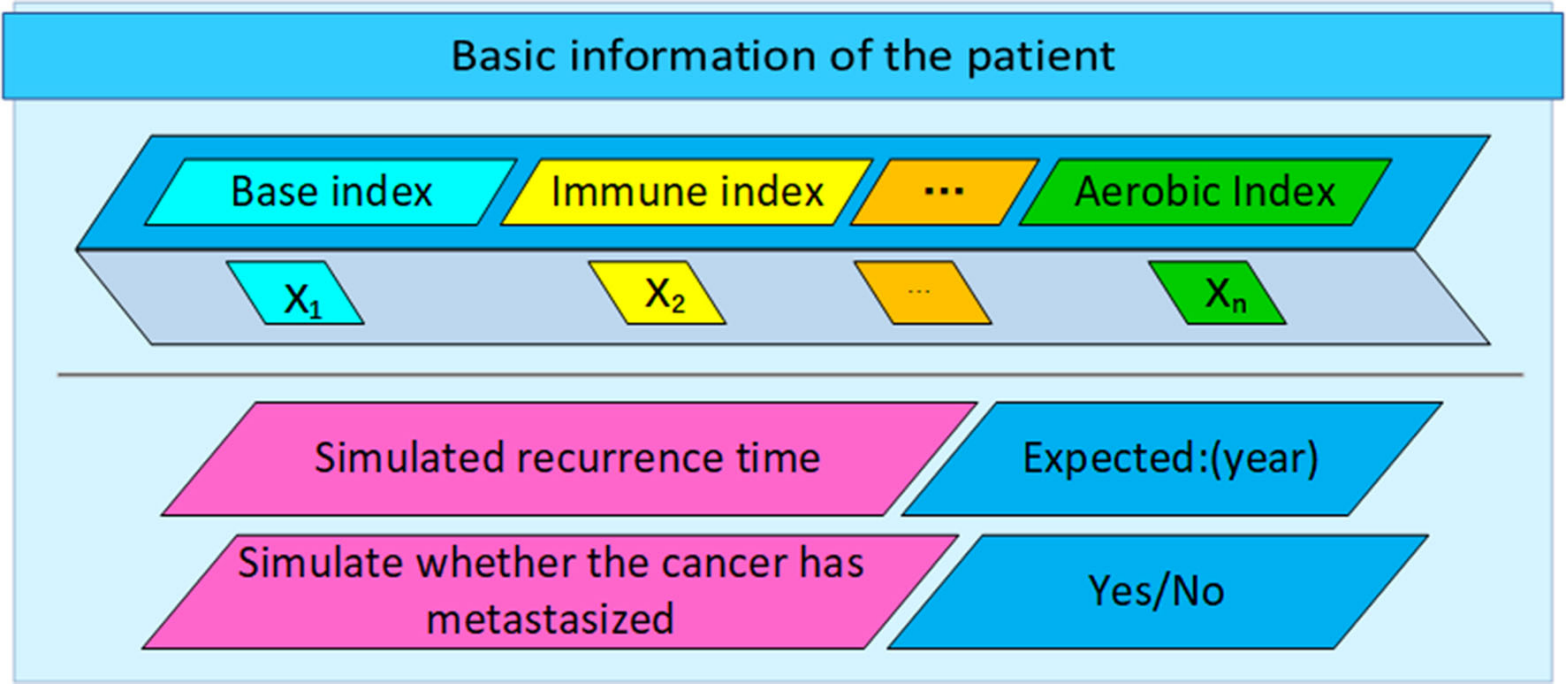
Interactive design of auxiliary diagnosis and treatment system.

From **Figure 6** above and **Figure 4**, it can be clearly seen that the parameters updated after multiple iterations of each participant through the federated learning framework based on the convolutional neural network-based auxiliary diagnosis model have obvious accuracy under 500 simulated simulation samples. Under the condition of an absolute error of ±6 months, although a certain proportion of noise data has been added to the participants to make each participant achieve homogeneity, the final experimental results indicate that the model simulates recurrence in various cancer patient’s The time accuracy can reach more than 90%. Through the comparison of the two methods, the federated learning enables the model to be trained locally on the basis of the patient data privacy and security, and the updated parameters are returned to update the overall model, which expands The patient data sample further improves the accuracy of the model. The intelligent diagnosis model can assist doctors in diagnosing cancer patients to a certain extent. It has application prospects and is of great significance for prolonging the lives of cancer patients. It also provides a way for doctors to diagnose patients. Effective reference and **Table 5** is a comparison of the above figure with the recurrence time and the pros and cons of the model. After that, we conducted corresponding experiments on whether the cancer cells metastasized when the patient’s cancer recurred, and used the diagnosis and treatment model to assist doctors in the diagnosis and rehabilitation of cancer patients. With the iteration and parameter return of the model under the federated learning framework, the recurrence position gradually stabilized at Around 90%, we finally learned through the federated learning model that the recurrence time is also in a different state with the changes of various indicators. This shows that regulating different indicators can help cancer patients to a certain extent. The interactive interface is designed to facilitate the doctor’s understanding, see **Figure 7** below.

**Table 5:** Model comparison.

Comparison of advantages and disadvantages	The amount of data	Safety	Accuracy (this experiment)	Participants
Unilateral modeling intelligent diagnosis model	Restricted	Low	65%-85%	Unilateral
A model for assisted diagnosis of cancer patients based on federated learning	Unrestricted	high	90%>	Multi-party joint

The auxiliary diagnosis system based on the federated learning framework has greatly increased the data sample size, increased the number of data iteration rounds, and guaranteed data security, greatly improving the accuracy of the model, and we have given an interactive design diagram, In order to facilitate the application of this model, through the accurate model and analysis of the correlation between various indications and cancer recurrence time, doctors can finally use the model and medical knowledge to intervene in the patient’s rehabilitation process, affect the patient’s rehabilitation indications, and improve patient survival rate.

## Conclusion

In the context of the continuous improvement of international privacy protection laws and regulations, data security gradually being valued by the public, and the prevalence of “data islands”, this article combines multiple hospitals with cancer patient data, and uses federated learning, neural networks, and localized differential privacy. Based on the homogenization of the center, a set of federated learning auxiliary diagnosis models for cancer patients was constructed, and the unilateral modeling machine learning assisted diagnosis models were compared. The accuracy and safety of the models have been greatly improved. The federated learning model effectively expand the training data of cancer patients, and protect the privacy and security of cancer patient’s’ data. This study only cooperated with three hospitals and one biological laboratory. Although the amount of data has increased significantly, there are still shortcomings for fatal cancers. It is expected that in the future, the number of participants will gradually increase. As the model is constantly updated, the cancer intelligent diagnosis and treatment system will eventually play its value.

Federated learning is one of the methods that can solve the current data security sharing problem. Compared with artificial intelligence methods that are widely used in all walks of life, such as unilateral machine learning and fuzzy systems, federated learning shows great advantages such as improving data privacy protection and expanding data volume. We have achieved gratifying results by applying it and applying it to the rehabilitation of cancer patients. It is expected to come, with continuous exploration and innovation. The phenomenon of “data islands” between various industries and enterprises will be broken, and data from all parties will be shared more reasonably and safely, allowing artificial intelligence to be applied to all corners of us, and we will continue to work on assisted diagnosis solutions for cancer patient’s The research, combined with more advanced mathematical models and machine learning models, and safer federated learning privacy protection methods, is to find the best solution for cancer patients on the premise of protecting patient data security and expanding cancer patient data.

## Data Availability Statement

The original contributions presented in the study are included in the article/supplementary material. Further inquiries can be directed to the corresponding author.

## Author Contributions

ZM contributed to the conception of the study, experimental part and writing the main body of the paper. MZ participated in the important experimental part of the paper. AY contributed to the construction of the overall thesis framework and the revision of some thesis content, as well as the follow-up communication of the thesis. DH is in charge of collation of experimental data and liaison with major hospitals. The rest of the authors are responsible for the alignment of data samples, construction of experimental models and other issues, and can be counted as authors with equal contributions. All authors contributed to the article and approved the submitted version.

## Funding

This work was supported by: 1. Key Science and Technology Project of Hebei Provincial Department of Education (North China University of Science and Technology, Project Number: JYG2020001); 2. Key Basic Research Project Fund of Hebei Provincial Department of Science and Technology (North China University of Science and Technology, Project Number: 20270902D); 3. Project funded by the Natural Science Foundation of Hebei Province. (North China University of Science and Technology, Project Number: E2021209024); 4. State Key Laboratory of Process Automation in Mining & Metallurgy and Beijing Key Laboratory of Process Automation in Mining & Metallurgy, the fund project number is: BGRIMM-KZSKL-2018-10.

## Conflict of Interest

The authors declare that the research was conducted in the absence of any commercial or financial relationships that could be construed as a potential conflict of interest.

## Publisher’s Note

All claims expressed in this article are solely those of the authors and do not necessarily represent those of their affiliated organizations, or those of the publisher, the editors and the reviewers. Any product that may be evaluated in this article, or claim that may be made by its manufacturer, is not guaranteed or endorsed by the publisher.
